# Survival of the Fittest: Addressing the Disparities in the Burden of Chronic Kidney Disease

**DOI:** 10.7759/cureus.9499

**Published:** 2020-07-31

**Authors:** Maxine L Nelson, Keri-Ann R Buchanan-Peart, Geraldine I Oribhabor, Rhutuja V Khokale, Ivan Cancarevic

**Affiliations:** 1 Internal Medicine, California Institute of Behavioral Neurosciences & Psychology, Fairfield, USA; 2 Obstetrics and Gynecology, California Institute of Behavioral Neurosciences & Psychology, Fairfield, USA; 3 Neurology, California Institute of Behavioral Neurosciences & Psychology, Fairfield, USA

**Keywords:** health care, mortality, morbidity, socioeconomic status, race/ethnicity, ckd, end-stage renal disease (esrd)

## Abstract

The prevalence of chronic kidney disease (CKD) is increasingly becoming recognized as a global health concern as well as a critical determinant of poor health outcomes. Decreased access to health care and low socioeconomic status (SES) worsen the adverse effects of biologic or genetic predisposition to CKD. All the studies used were retrieved using the PubMed database. The literature suggests that in developing and developed countries, lower SES is inversely proportional to CKD. It shows an inconsistent relationship between CKD and race; that is, there may or may not be a relationship between these two variables. In the United States (US), the prevalence of the early stages of CKD is similar across different racial/ethnic groups. However, the preponderance of end-stage renal disease (ESRD) is higher for minorities than their non-Hispanic white counterparts.

Further investigation is required to understand the role of racial disparities and CKD as well as to understand the significant difference seen in the incidence when progressing from CKD to ESRD. It is necessary to recognize how lower SES and racial/ethnic disparity may result in the impediment of appropriate disease management. A possible approach is the use of the biopsychosocial model, which integrates biological, individual, and neighborhood factors. A practical method of providing appropriate care to these populations will require economically feasible prevention strategies as well as extending the scope of dialysis by the implementation of cheaper alternatives.

## Introduction and background

 

“Health inequalities and the social determinants of health are not a footnote to the determinants of health. They are the main issue." Michael Mormot.

Chronic kidney disease (CKD) is characterized by severe, irreversible kidney damage in which there is a reduction in glomerular filtration rate (GFR) <60 ml/min per 1.73 m^2^ or a urinary albumin-to-creatinine ratio >30 mg/g [1]. CKD may progress to end-stage renal disease (ESRD), which is characterized by GFR <15 ml/min; generally, these patients require hemodialysis and kidney transplantation [2]. In the United States (US), kidney disease is the ninth leading cause of death [3]. Studies estimate that 80,000 persons are diagnosed with CKD annually, and approximately 19 million persons are living with the condition in the US. CKD progression is associated with numerous severe complications, such as hyperlipidemia, anemia, metabolic bone disease, and an increased incidence of cardiovascular disease [4]. The prevalence of CKD is increasingly becoming recognized as a global health concern as well as a core determinant of poor health outcomes [5]. Compared with the general population, age-adjusted mortality rates are six to eight times higher for those with ESRD than the general population [6]. Studies have shown compelling evidence that disadvantaged communities, that is, those from low resource, racial, and minority ethnic communities, experience a more significant increase in the burden of unrecognized and untreated CKD [5]. The incidence of ESRD is 3.4 times higher in blacks compared with whites in the US [6]. The prevalence of CKD risk factors, genetic predisposition, low socioeconomic status (SES), and inequalities in access to care is responsible for the increased incidence [7]. Living in a low-income or racially segregated area predicted worse outcomes for persons commencing dialysis [6,8]. Worldwide, approximately 1.2 billion people live in extreme poverty [5]. Poverty negatively impacts health-seeking behaviors and access to health care, and, by extension, contributes to health care disparities. Deceased access to health care and low SES worsen the adverse effects of biologic or genetic predisposition to CKD.

There are widespread health disparities in CKD that require sincere efforts towards elimination [9]. For decades, the differences in racial/ethnic and SES are acknowledged; however, very little work is being done to mitigate such factors [8]. Previous studies have shown that the elimination of health disparities is a national priority; however, there are very few interventions that exhibit a reduction in this disparity among patients with CKD [10]. More recent studies highlight the origin of these disparities and emphasize the importance of future research to implement policy changes [8].

Racial/ethnic minorities and lower socioeconomic class have a disproportionate burden of CKD and have worse outcomes even in developed countries, therefore suggesting that more investigation should be conducted beyond the traditional risk factors of CKD and its complications [5]. The integral steps that should be taken by health care providers and public health practitioners involve treating and controlling risk factors for the disease to prevent a further upward trend [3]. The development of pioneering health care strategies, innovative policy interventions, and novel policy interventions requires eliminating this disparity associated with CKD [9]. Findings propose that CKD patients with limited education or lower income should be targets for early intervention to limit disability and further loss of income, both of which can potentially worsen the outcomes of CKD patients [11]. Continued work is required to identify modifiable environmental, social, and behavioral factors for targeted interventions as these will improve the care and prognosis of CKD patients [8]. 

This literature review aims to highlight the disparities of SES and racial/ethnic minorities in CKD and offer insight into future interventions at addressing CKD as a global burden. 

## Review

SES and CKD

There is an inverse relationship between individual SES and progressive kidney disease [12]. SES modifies well-known risk factors that contribute to the development of kidney disease [13]. Crews et al. found that after adjustment for demographics, insurance status, and comorbid disease, there was an independent relationship between low SES (defined as <125% of the national level) and CKD with 59% greater odds (odds ratio [OR] 1.59 95% confidence interval [CI] 1.27-1.99) [14]. Similarly, Merkin et al. used Cox proportional hazards models to demonstrate a relationship between SES area quartile and progressive CKD [15]. SES area quartile was characterized using income, wealth, education, and occupation, and progressive CKD was defined as hospitalization for CKD or creatinine elevation 0.4 mg/dL over a four- to seven-year follow-up. It was demonstrated that living in the lowest SES area quartile compared to the highest was associated with a 50% increased risk of progressive CKD which was defined as hospitalization for CKD or creatinine elevation 0.4 mg/dL over a four- to seven-year follow-up. In the Atherosclerosis Risk in Communities (ARIC) Study, the relationship between kidney disease and SES was evaluated by Shoham et al. [16]. The study showed that the adjusted OR of CKD for persons belonging to the working class compared to non-working class was 1.4 (95% CI, 1.0-2.0) in whites and 1.9 (95% CI, 1.1-3.0) in African Americans [16]. In a study by Fedewa et al., compared to higher-income participants, low-income participants had an increased adjusted hazard of mortality (HR, 1.58; 95% CI, 1.24-2.00) [17]. They also demonstrated that regardless of race, low income was associated with all-cause mortality (HR, 1.53; 95% CI, 1.18-1.99 among blacks and HR 1.38; 95% CI, 1.10-1.74 among whites). Additionally, in the Jackson Heart Study, Bruce et al. concluded that opulent African Americans had a 41% lower prevalence of CKD than their less opulent counterparts [18]. In a systematic review and meta-analysis, Vart et al. concluded that low estimated glomerular filtration rate (eGFR) was associated with low SES (OR, 1.41; 95% CI,1.21-1.62), high albuminuria (OR, 1.52; 95% CI, 1.22-1.82), low (eGFR/high albuminuria (OR, 1.38; 95% CI, 1.03-1.74), and renal failure (OR, 1.55; 95% CI, 1.40-1.71) [19].

Overall, it is clear that SES is strongly related to the incidence and prevalence of CKD and ESRD. Persons subjected to adverse living conditions are at an increased risk of CKD irrespective of race. This risk is present despite adjusting for independent lifestyle factors such as hypertension and diabetes. Undoubtedly, the relationship between SES and CKD is quite complicated and involves multifactorial and multilevel mechanisms. The efforts made to comprehend these health disparities should take these effects into account. A possible approach is the use of the biopsychosocial model, which integrates the use of biologic, individual, and neighborhood factors.

Race and CKD

In the US, there is an independent association between racial and ethnic disparities and poor health outcomes in CKD [20]. Similarly, there is a strong association between racial and ethnic inequality and lower SES. The prevalence of poverty among African Americans is more significant than that of whites, thereby contributing to racial disparity [21]. Additionally, a lot of the increased risk of non-diabetic kidney disease in persons of African descent may be explained by the apolipoprotein 1 (APOL 1) gene variant, which is common in African descent and often absent in people of European descent [22]. Homozygosity for one of the APOL1 risk variants is associated with elevated albuminuria, low GFR, and faster progression of CKD. Vart et al. indicated in a meta-regression analysis study that race is possibly related to the degree of association between low SES and CKD (P value = 0.001) [19]. Additionally, they demonstrated that the relative risk (RR) of CKD in low-SES people was 58% higher in African Americans (RR, 1.58; 95% CI, 1.33-1.84) and 91% higher in whites (RR, 1.91; 95% CI, 1.47-2.35) when compared with their high-SES peers. Fedewa et al. in the Reasons for Geographic and Racial Differences in Stroke (REGARDS) study highlighted that participants with prevalent CKD stage 3 or 4 had a higher adjusted hazard of mortality (HR, 1.30; 95% CI, 1.02-1.65) independent of income, seen in black participants compared to whites [17]. The National Health and Nutrition Examination Survey (NHANES) data demonstrated that black Americans with and without diabetes have 2.8 and 2.2-fold greater odds of having abnormal albuminuria versus white Americans, respectively [22]. Crews et al. concluded that there was no association between race and CKD (OR, 1.05; 95% CI, 0.57-1.96) [14]. Conversely, the odds of advanced CKD (eGFR <30 mL/min/1.73 m^2^) was higher in African Americans. Stratification by race, however, showed that low SES was associated with CKD in African Americans (OR, 1.91; 95% CI, 1.54-2.38), but not whites (OR, 0.95; 95% CI, 0.58-1.55; P = 0.003). Hsu et al. concluded that there was no difference in the prevalence of chronic renal insufficiency (CRI) (GFR 15 to 59 ml/min per 1.73 m^2^) in a comparison of blacks and white adults, with results showing 2,060 versus 2,520 per 100,000, respectively (P = 0.14) [23]. In 1991, for every 100 blacks with CRI, there were five new cases of ESRD development in 1996. For every 100 whites, only one case of ESRD developed (RR, 4.8; 95% CI, 2.9-8.4). After adjusting for age, gender, and diabetes, the increased risk for blacks versus whites was moderately affected. Shah et al. demonstrated that after the initiation of dialysis, the adjusted risk of dying at one year was lower in blacks (OR, 0.73; 95% CI, 0.72-0.74), Hispanics (OR, 0.64; 95% CI, 0.63-0.65), Asians (OR, 0.55; 95% CI, 0.53-0.56), and Native Americans (OR, 0.67; 95% CI, 0.63-0.71) as compared to whites [24]. Noori et al. demonstrated that there was a strong association between higher levels of C-reactive protein (CRP) and interleukin-6 (IL-6) and an increase in the adjusted mortality risks of 799 African American and white dialysis patients followed over six years [25]. There was a 2.4 and 4.1 higher risk of death in African Americans and whites, respectively, using the highest quartile of IL-6 versus the lowest.

The results indicate that there is a complex interplay of factors between race and CKD. Assessment of this relationship has yielded inconsistent results, which may potentially be related to the differences in the populations examined. The meta-analysis of SES, race, and CKD demonstrated that a significant relationship existed between such variables. The REGARDS study concluded that their black participants with advanced stage CKD had worse outcomes. Additionally, the NHANES study denoted that generally, there is a greater incidence and prevalence of albuminuria in black than in white individuals. A postulation for these findings could be the presence of the APOL 1 gene variant seen in individuals of black ancestry.

Conversely, the study conducted by Crews et al. indicated no association between race and CKD; however, the likelihood of advanced CKD was in African Americans [14]. The study showed a significant relationship between low SES and CKD in African Americans but not whites. The prevalence of the early stages of CKD is similar across different racial/ethnic groups (Table 1).

**Table 1:** Relative Prevalence of CKD by Race/Ethnicity CKD, chronic kidney disease

Race/Ethnicity	Relative Prevalence of CKD 1-3
White	1
Hispanic	0.58
Black	0.92

However, the preponderance of ESRD is higher for minorities than their non-Hispanic white counterparts (Table 2).

**Table 2:** : Relative Prevalence of ESRD by Race/Ethnicity ESRD, end-stage renal disease

Race/Ethnicity	Relative prevalence of ESRD
White	1
Asian	1.6
Native American	1.96
Hispanic	1.98
Black	4

The study by Shah et al. concluded that even though the likelihood of death is higher in African Americans with CKD than in non-Hispanic individuals with CKD, once ESRD is established, there is a reversal in the trend [24]. The racial/ethnic differences in inflammation highlighted by Noori et al. may be the reason for the unexpected difference in survival between African American and white patients on dialysis [25]. Further investigation is required to understand the role of racial disparities and CKD as well as to understand the significant difference seen in the incidence when progressing from CKD to ESRD. Previous studies have been unable to explain the complex relationship between CKD and race indicating that age, comorbidities, and SES play a critical role. They have also indicated that the key to understanding the relation is to focus on the progression from CKD to ESRD rather than focusing on the prevalence of CKD across racial groups. 

CKD in developed countries

The rates of treated ESRD are higher in ethnic minority groups in the United Kingdom (UK) [26]. A similar pattern is also seen in Singapore, where the prevalence of CKD is higher among Malays and Indians compared to the Chinese. This excess risk is due to socioeconomic and behavioral factors that account for 70%-80%. Krishnasamy and Gray reported that in Australia, the mortality for the most advantaged quartile was 102.4/1,000 person-years (95% CI, 98.0-106.9) compared with 110.7/1,000 person-years (95% CI, 105.8-115.7) in the disadvantaged quartile [27]. Fraser et al. demonstrated that in England age sex-adjusted CKD 3-5 was associated with lack of qualifications (OR, 2.27; 95% CI, 1.40-3.69), low income (OR, 1.50; 95% CI, 1.02-2.21), and renting tenure (OR, 1.36; 95% CI, 1.01-1.84) [28]. They also showed that albuminuria is associated with several SES measures on full adjustment: low income (OR, 1.55; 95% CI, 1.14-2.11), no vehicle (OR, 1.38; 95% CI, 1.05-1.81), renting (OR, 1.31; 95% CI, 1.03-1.67), and most deprived area-level quintile (OR, 1.55; 95% CI, 1.07-2.25). Garcia-Garcia et al. reported that the rate of ESRD experienced by Canadian First Nations is 2.5-4 times higher than the general population [5]. Akrawi et al. demonstrated that in Sweden, there was a significant relationship between ESRD and neighborhood deprivation (age-adjusted OR, 1.45; 95% CI, 1.34-1.56 in men and OR, 1.59; 95% CI, 1.44-1.75 in women) [29]. The ORs for ESRD in men and women living in the most underprivileged neighborhoods remained significantly elevated, when adjusting for individual-level sociodemographic factors and age (OR, 1.25; 95% CI, 1.15-1.35 in men and OR, 1.30; 95% CI, 1.17-1.44 in women). Shen et al. demonstrated that in China, patients with CKD were farmers with limited access to health care facilities and were of a lower educational level [30].

Poor access to health care and low SES contribute to the disparities seen in CKD, even in developed countries. The incidence of ESRD is significantly higher in the less advantaged populations. In Australia, where access to health care is universal, low SES had an adverse effect on the survival rate of dialysis patients. This finding indicates that there is a delicate interplay of nature versus nurture factors existing. Similar to environmental and genetic components, social determinants of health play a significant role in the management of CKD. As a result, it is necessary to recognize these factors in patients and see how these may be mitigated to prevent the impediment of appropriate disease management.

CKD in developing countries

There is a variety of poverty-related factors that play a significant role in the increase of CKD in low-income countries [5]. These include infectious diseases due to poor sanitation, lack of safe water supply, and environmental pollutants. It is projected that the number of individuals receiving renal replacement therapy worldwide will increase to 5.4 million by 2030 and will predominantly occur in countries of Asia and Africa. Garcia-Garcia et al. demonstrated that there was a twofold to threefold higher CKD prevalence among the poor compared to the general population in Mexico [5]. Baretto et al. reported that in Brazil, compared to those individuals with a university degree, having high school (OR, 1.15; 95% CI, 1.00-1.34) or elementary education (OR, 1.23; 95% CI, 1.03-1.47) increased the likelihood for CKD [31]. It was shown by Tannor et al. that in Ghana, individuals with low-income status had moderate to advanced CKD and had a low quality of life [32]. 

Overall, the studies have shown a similar trend that both worldwide factors and population-specific issues contribute to the increased burden of CKD. In developing and developed counties, lower SES is inversely proportional to CKD. A practical method of providing appropriate care to these populations will require economically feasible prevention strategies as well as extending the scope of dialysis by the implementation of cheaper alternatives.

## Conclusions

There is a disproportionate burden of CKD experienced by racial/ethnic minorities and individuals of lower SES. There is an inverse relationship between individual SES and progressive kidney disease. An increased burden of CKD is in developing and developed counties, for which the cause is lower SES. The relationship between CKD and race is less straightforward and involves a complex interplay of factors. Further investigation is required to understand the role of racial disparities and CKD. The provision of appropriate treatment to these populations should integrate the use of the biopsychosocial model.
